# Biomechanical influence of numerical variants of lumbosacral transitional vertebra with Castellvi type I on adjacent discs and facet joints based on 3D finite element analysis

**DOI:** 10.1016/j.bonr.2025.101831

**Published:** 2025-02-20

**Authors:** Tongxin Zhu, Zhangyan Xu, Dan Liu, Wei Zeng, Yongliang Pu, Haitao Yang

**Affiliations:** Department of Radiology, the First Affiliated Hospital of Chongqing Medical University, 1 Youyi Road, Yuzhong District, Chongqing 400016, China

**Keywords:** Finite element analysis, Lumbosacral transitional vertebrae, Spinal curvature, Back pain

## Abstract

**Objectives:**

To investigate the effect of lumbosacral transitional vertebra (LSTV) on the biomechanical properties of adjacent discs and facet joints based on geometrically 3D personalized FEA.

**Methods:**

A total of 45 individuals who underwent low dose whole body CT scans were retrospectively included and equally divided into 23, 24, and 25 presacral vertebrae (PSV) groups. Three-dimensional Finite Element computational models of normal and number-variant sub-types of LSTV were created. The biomechanical parameters, including the range of motion (ROM), the intervertebral disc pressure (IDP), and facet joint forces (FJF), were all evaluated to determine the biomechanical effects. IDP was equally divided into anterior (AIDP), middle (MIDP) and posterior (PIDP) parts along the short axis of the intervertebral disc.

**Results:**

During extension, the 23 PSV group exhibited significantly higher von Meiss stress in the upper intervertebral disc compared to the 24 and 25 PSV groups (*P* = 0.003), indicating concentrated stress in the upper lumbar region and an increased the likelihood of localized disc degeneration over time. Furthermore, the 23 PSV group exhibited a larger ROM (3.28°) than the 25 PSV group (1.40°) (*P* = 0.011), implying greater segmental mobility and possible instability in the transitional segment. During flexion, the 25 PSV group showed higher stress in the lower intervertebral disc and a larger ROM than the 23 and 24 PSV groups; however, the differences were not significant (*P* > 0.05).

**Conclusions:**

The increased stress distribution and ROM in the upper disc of the transitional segment were only found in the 23 PSV sub-type of Castellvi type I LSTVs during extension, but not in the 25 PSV sub-type, which may help to further understand the impact of LSTV on the surrounding structures.

## Highlights

• Number-variant of LSTV changed stress distribution on adjacent structures.
• The increased stress of upper disc was only found in lumbar sacralization subtype.
• Extension might be a potential factor leading to discal degeneration in LSTV.
• Number-variant of LSTV showed no effects on the stress of facet joints.

## 1 Introduction

Lumbosacral transitional vertebrae (LSTV) are a common morphological developmental malformation, with a prevalence of 10.0 %–18.1 % in healthy people and 4.6 %–35.6 % in people with low back pain (Khalsa et al., 2018; Nardo et al., 2012; Tang et al., 2014; Yokoyama et al., 2016). LSTV can be divided into lumbar-sacral vertebrae and sacral-lumbar vertebrae according to the number of variations with incidences of 6.7 % and 2.7 % respectively (Rabau et al., 2021). The Castellvi classification categorizes LSTV into four types (I to IV) based on the degree of fusion or articulation between the lowest lumbar vertebra and the sacrum (Castellvi et al., 1984). The exact etiology of LSTV remains unclear and may be related to genetics. HOX genes are reported to be responsible for the development of the lumbosacral vertebrae, and they play an important role in the segmentation of the axial skeleton during embryogenesis (Carapuço et al., 2005; Wellik and Capecchi, 2003). Studies have reported that there are strong latent relations between LSTV and clinical features such as low back pain, intervertebral disc degeneration, and neuropathy. Bertolotti syndrome is used to describe the association between LSTV and low back pain (Benvenuto and Benvenuto, 2018; Luoma et al., 2004; Quinlan et al., 2006). The presence of LSTV changes the biomechanics and alignments of the spino-pelvic complex and local stress and shearing force of lumbo-sacral region, which can accelerate the degeneration of the adjacent intervertebral disc, especially in young people (Luoma et al., 2004; Quinlan et al., 2006). However, there are also some controversial findings. A cross-sectional MRI study reported no association between LSTV and low back pain in middle-aged individuals (Luoma et al., 2004). The current mainstream opinion considers that the existence of LSTV can protect the transitional segment, and aggravate the damage to the upper section of the intervertebral disc (Farshad-Amacker et al., 2015; Fitzgerald and Newman, 1976; Frymoyer, 1994; Mahmoodkhani et al., 2024). However, several studies reported that similar results were not found in Castellvi type III-IV of LSTV and middle-aged subjects (Castellvi et al., 1984; Luoma et al., 2004). The influence of LSTV on the adjacent discs of transitional segments remains controversial. This may be due to a lack of detailed understanding of the impact of LSTV sub-types on the mechanism of the lumbosacral region. Thus far, the related anatomic geometry and biomechanical changes of the different sub-types of LSTV on adjacent structures remain unclear, which may be essential to understanding the pathophysiological mechanism, personalized treatment and prevention of LSTV-associated early onset spinal degeneration.

The finite element analysis (FEA) is a mathematic engineering resource utilized to calculate the stress force and strain distribution of complex structures and become a prevailing research approach in biomechanical orthopedics. FEA has been proven to hold great value in simulating the various states of the spine and exploring the mechanism of spinal injury and pathology, and treatment effectiveness (Elmasry et al., 2016; Wang et al., 2006; Wei et al., 2021; Wilcox, 2006; Yamamoto et al., 1989). Recently, a three-dimensional (3D) FEA study reported there were decreased motion range and increased stress at the side of L5-S1 pseudoarthrosis, and corresponding reduced stress of the ipsilateral facet joint. However, this study applied only one LSTV and control subjects to establish models, which was not adequate for evaluating of statistical significance of the outcomes (Mahato et al., 2019). The purpose of this study was to investigate the effect of Castellvi type I LSTVs number-variant sub-types on the biomechanical properties of adjacent discs and facet joints based on geometrically 3D personalized FEA.

## 2 Materials and methods

### 2.1 Individuals and acquisition of CT images

Institutional review board approval was obtained for this retrospective study. The subjects were enrolled from the data published in our previous studies about quantitative imaging analysis of LSTV (Zhou et al., 2022). Subjects with complete LSTV numeric variation confirmed by the gold standard of counting caudally from C2 on the whole spine PET-CT image. The exclusion criteria were as follows: spinal deformities including butterfly vertebra, hemivertebra and kyphosis, previous history of spinal fracture and surgery, spine with severely degenerative changes, cases not clearly showing vertebra or disc, numeric variation combined transverse process pseudoarthrosis (Castellvi type II-IV). Four lumbar vertebrae were defined as 23 presacral vertebrae (PSV), corresponding to L5 complete sacralization. Six lumbar vertebrae were defined as 25 PSV, corresponding to S1 complete lumbarization. Finally, there were 45 subjects based on propensity score matching (PSM) to match the age, sex, and BMI, and divided into three groups including the normal group (24 PSV) (case = 15; 7 males, 8 females; age range, 32–55 years; median age, 49.0 years), 23 PSV (case = 15 lumbar sacralization; 7 males, 8 females; age range, 41–64 years; median age, 53.0 years) and 25 PSV group (case = 15 sacral lumbarization; 7 males, 8 females; age range, 35–60 years; median age, 53.0 years) with a tolerance of 0.0001.

The PET/CT images were acquired on a PET/CT scanner (Philips Gemini TF 64 PET/CT scanner). The low-dose CT images were obtained with a standardized protocol of 100 mA, 120 kV, matrix size of 512 × 512, and a slice thickness of 2 mm.

### 2.2 Geometric modeling

A total of 384–510 DICO whole spine CT images from the upper edge of C2 to the sacrum with a slice spacing of 2.0 mm were collected for each subject. Using the CT scan dataset, the target area was isolated in Mimics 21.0 (Materialise, Belgium), and a 3D geometric model of the lumbosacral spine was created by adjusting the gray threshold and manually refining the 3D reconstruction. The 3D models from Mimics were then exported to Geomagic 2021 (Geomagic, USA) in STL format for geometric routing and surface smoothing enhancements. Reverse engineering was conducted in Solidworks 2021 (Altair, USA) to develop a 3D disc contour model. The meshes and material properties were defined in Ansys 2021 R1 (Simula, USA), where finite element analysis was carried out.

### 2.3 Mesh delineation and material property assignment for finite element models

**Fig. 1:**
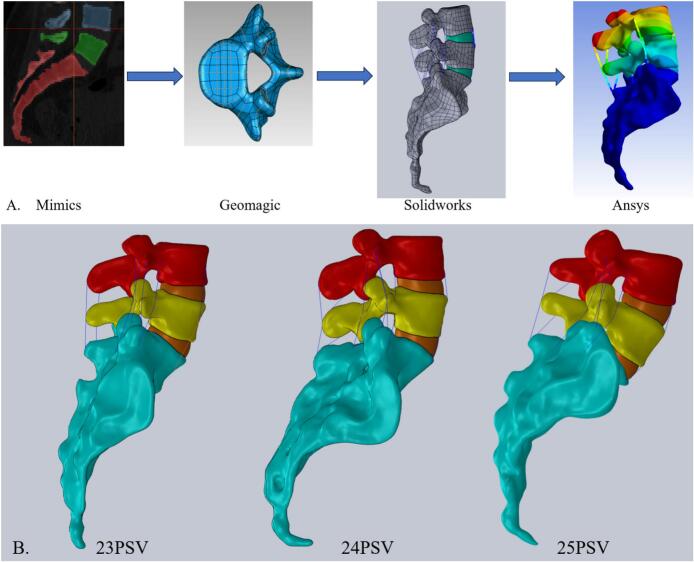
Finite element models of lumbosacral vertebrae A. Flow chart of lumbosacral region finite element model establishment. B. Lateral view of finite element model of lumbosacral vertebrae with different number variation.

The complete nonlinear finite element model shown in (Fig. 1) included cortical bone, cancellous bone, posterior structures, intervertebral discs, endplates, and ligaments. Cortical bone and cancellous bone are considered orthogonal anisotropic materials (Schmidt et al., 2007). Cortical bone thickness was set to 1 mm (Zhang et al., 2022). The ligamental models included seven separate ligaments as follows: the capsular ligament (CL), intertransverse ligament (ITL), supraspinous ligament (SL), interspinous ligament (ISL), ligamentum flavum (LF), anterior longitudinal ligament (ALL), and posterior longitudinal ligament (PLL). All the ligaments were modeled as tensile-only truss elements (Polikeit et al., 2003). Subsequently, the corresponding material properties were given to the bone, ligament, cartilage, intervertebral disc and other elements in the model, so as to restore the physiological conditions of each tissue material as much as possible and improve the reliability of the model, as shown in (Table 1), and the material parameters were referred to the relevant literature (Bae et al., 2013; Liu et al., 2021; Shi et al., 2014; Wen et al., 2020; Zhang et al., 2022; Zhu et al., 2022).

**Table 1:** Material properties of the model.

Component	Young's modulus (MPa)	Poisson ratio	Cross-sectional area (mm2)
Vertebral body	/	/	/
Cortical bone	12,000	0.3	/
Cancellous bone	100	0.2	/
Endplate	24	0.3	/
Nuclear pulposus	1	0.499	/
Annulus fibrosus	4.2	0.45	/
Facet joints	10.5	0.45	/
Ligaments	/	/	/
Anterior longitudinal ligament	20	0.3	63.7
Posterior longitudinal ligament	20	0.3	20
Interspinous ligament	11.6	0.3	40
Supraspinous ligament	15	0.3	30
Intertransverse ligament	58.7	0.3	3.6
Capsular ligament	32.9	0.3	60
Ligamentum flavum	19.5	0.3	40

### 2.4 Boundary conditions and contact settings

Stress load was defined to add 500 N axial following loads (AFL) on the upper vertebral body, and the S1 vertebral body was completely fixed on all degrees of freedom under the surface. Moreover, an additional 7.5 N-m moment was imposed on both flexural and extensional motion separately. Finally, the biomechanical parameters from the upper to lower transitional lumbosacral segment of the simulated cases were calculated and compared, including a range of motion (ROM), intervertebral disc pressure (IDP), facet joint forces (FJF). IDP was equally divided into anterior (AIDP), middle (MIDP) and posterior (PIDP) parts along the short axis of the intervertebral disc.

### 2.5 Statistical analysis

Statistical analyses were performed with the SPSS statistical software program (version 26.0; SPSS). The Shapiro-Wilk test was used to check the normal distribution of all measured parameters in the three groups. The normal distribution parameters were analyzed by one-way ANOVA to compare the differences in the parameters among the three groups. Kruskal-Wallis univariate analysis of variance was performed for non-normal distribution parameters. For multiple comparisons between the three groups, Bonferroni correction was used, and *P* < 0.05/n (*n* = 3) was considered statistically significant. With the exception of Bonferroni-adjusted statistical analyses, *P* < 0.05 was considered statistically significant.

## 3 Results

### 3.1 Comparison of the lumbar curvature, disc volume and validation of the model

**Fig. 2:**
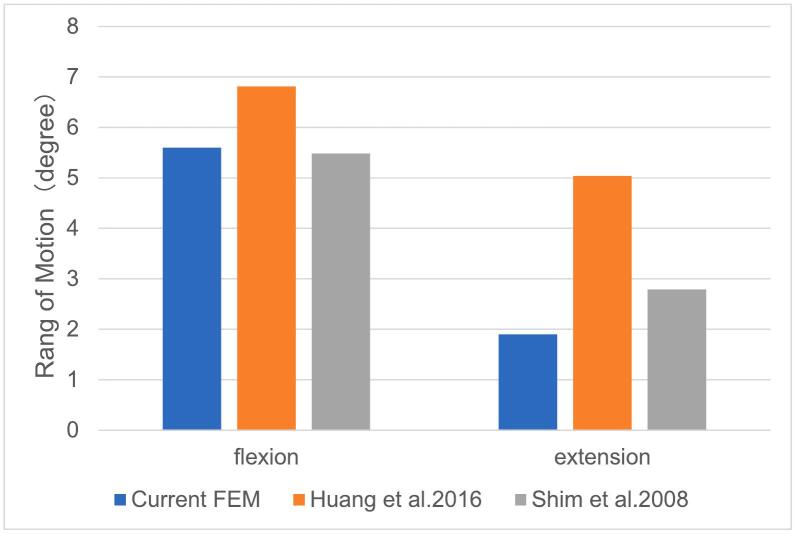
Comparison of flex-extension ROM in vitro and finite element model. The comparison shows that the ROM is consistent with those experimental measurements.

Among the three groups, the lumbar curvature showed a generally increased trend following the increment of lumbar numeration. However, no significant difference in the lumbar lordosis angles among them (*P* = 0.06). The upper disc volume of the transitional segment was generally greater than the lower disc volume in each group, with the smallest volume at the lower disc of 24 PSV, however, there were no significant differences among them (*P* = 0.771, 0.686). (Table 2). The normal 24 PSV model has a ROM of 5.6° and 1.9° in flexural and extensional states which were similar to the previous vitro and FEA studies (Renner et al., 2007; Shim et al., 2008). The data of this model could be identified as reliable to ensure the authenticity and accuracy of the experimental data. (Fig. 2).

**Table 2:** The general demographic characteristics, BMI, lumbar lordosis and adjacent disc volume of lumbo-sacral segment in three groups.

	23(*n* = 15)	24(n = 15)	25(n = 15)	*P* value
Gende, n(%)				
Male	7(46.7 %)	7(46.7 %)	7(46.7 %)	
Female	8(53.3 %)	8(53.3 %)	8(53.3 %)	
Age (years), n	49.00(39.5，53.50)	53.00(50.00，55.50)	53.00(46.5，58.00)	0.083
Height(m)	1.60(1.51，1.64)	1.60(1.56，1.65)	1.61(1.57，1.66)	0.839
Weight(kg)	58.00(50.50，66.50)	60.00(54.50，65.00)	60.00(55.50，63.50)	0.393
BMI(kg/m^2^)	22.66(21.97，25.65)	22.77(22.05，23.39)	23.01(20.28，24.19)	0.846
Lumbar lordosis (°)	44.41(40.35，47.90)	48.58(33.49，62.34)	57.10(50.57，62.13)	0.063
Upper disc volume(mm^3^)	7944.00(7262.75，10,794.00)	7701.30(6257.90，10,217.60)	7765.00(7081.30，10,547.00)	0.771
Lower disc volume(mm^3^)	7038.70(6485.40，9432.20)	6583.60(5514.00，8088.01)	6596.10(632,912，77,988.25)	0.686

### 3.2 Compared the AFL of IDP and FJF on the neutral position

**Fig. 3:**
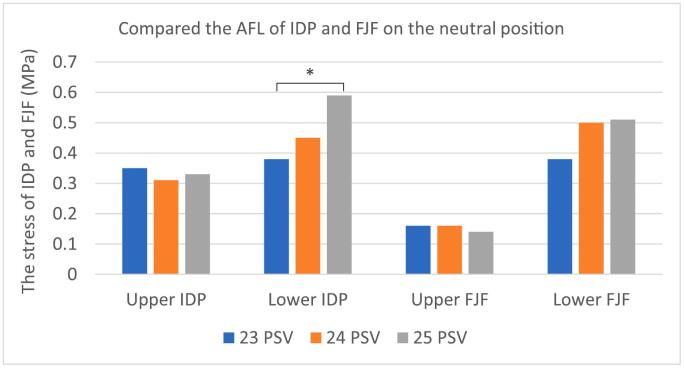
Comparison of IDP and FJF among the normal group (24 PSV), lumbar sacralization group (23 PSV) and sacralization group (25 PSV) on the neutral position. *P < 0.05.

**Fig. 4:**
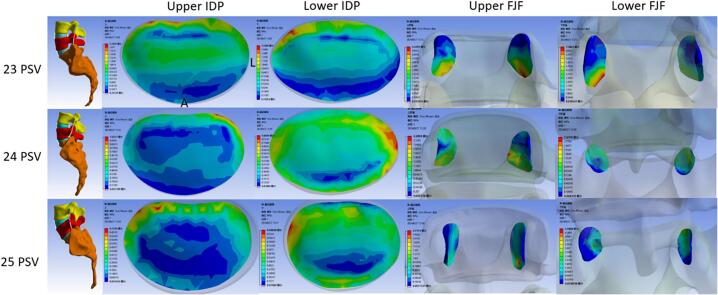
Stress nephogram of IDP and FJF from upper to lower transitional lumbosacral segment in three groups on neutral position.

Among the three groups, stress generated by AFL on the disc was generally increased from the upper to lower transitional lumbosacral segment in each group. There was no significant difference in the von Meiss stress intensity at the upper disc level among the three groups (*P* = 0.90). However, significant differences were found at the lower disc level of the transitional segment among the three groups (*P* = 0.014). The stress in the 25 PSV group (0.59 MPa) was significantly higher than that of the 23 PSV group (0.38 MPa) (*P* = 0.004). The von Meiss stress of the lower disc of the transitional segment in the 25 PSV spine was concentrated in the middle (0.61 MPa) and posterior (0.74 MPa), which was significantly higher than that of the 23 and 24 PSV (*P* = 0.007, 0.043). (Table 3, Fig. 3, Fig. 4).

**Table 3:** The finite element analysis results of the three groups of lumbar vertebrae under axial following loads were compared - multiple samples Kruskal-Wallis test.

		23	24	25	P value	P^a^	P^b^	P^c^
Upper disc pressure(MPa)	IDP	0.35	0.31	0.33	0.900			
	AIDP	0.34	0.32	0.34	0.730			
	MIDP	0.27	0.23	0.26	0.578			
	PIDP	0.44	0.45	0.41	0.980			
Lower disc pressure(MPa)	IDP	0.38	0.45	0.59	0.014	1.0	0.013	0.155
	AIDP	0.39	0.44	0.50	0.083			
	MIDP	0.31	0.38	0.61	0.005	1.0	0.007	0.043
	PIDP	0.58	0.58	0.74	0.038	1.0	0.039	0.070
Upper FJF(MPa)		0.16	0.16	0.14	0.596			
Lower FJF(MPa)		0.38	0.50	0.51	0.301			

Pa refers to the *p*-value of 23 compared to 24.

Pb refers to the *p*-value of 23 compared to 25.

Pc refers to the *p*-value of 24 compared to 25.

For articular cartilage stress of facet joints, there was no significant difference in the von Meiss stress at the upper segment among the three groups (*P* = 0.596). In the lower section, the articular cartilage stress increased following the rise of PSV number. However, there was also no significant difference among them (*P* = 0.301).

### 3.3 Compared the flexural moment of IDP, FJF and ROM on the flexural position

At the upper disc level of the transitional segment, there was no significant difference in intervertebral disc stress among the three groups (*P* = 0.161). At the lower disc level, the maximum disc stress was found at 25 PSV (0.98 MPa), but also no significant difference among them after Bonferroni correction. In flexion, the stress of the anterior part of the disc increased, and corresponding the stress discrepancy between the anterior and posterior parts decreased in each group, but still no significant difference among the three groups. The stress of the anterior and middle parts at the lower disc level in 25 PSV was significantly greater than that at 23 PSV (*P* = 0.028, 0.043).

For articular cartilage stress, there was no significant difference at the upper FJF of the transitional segment among the three groups. In the next section, the stress of the articular cartilage in the 25 PSV group was the biggest, but still no significant difference (*P* = 0.148).

**Fig. 5:**
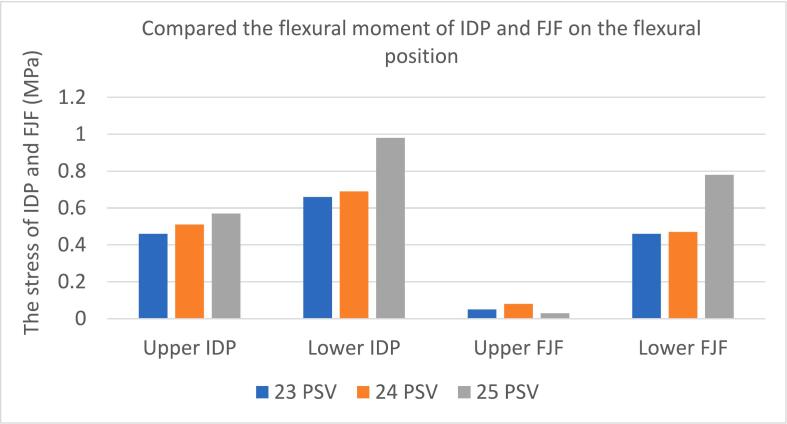
Comparison of IDP and FJF from upper to lower transitional lumbosacral segment in three groups on the flexural position. *P < 0.05.

**Fig. 6:**
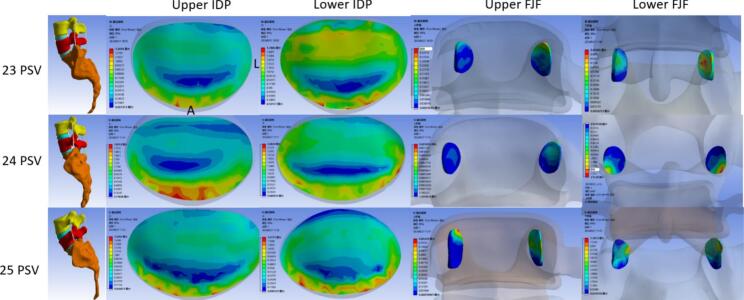
Stress nephogram of IDP and FJF from upper to lower transitional lumbosacral segment in three groups of lumbar vertebrae on the flexural position.

As the number of PSV increased, the ROM of the lumbar spine in flexion was also extended. The 25 PSV showed the largest ROM (6.29°) while there was no significant difference among the three groups (*P* = 0.116). (Table 4, Fig. 5, Fig. 6).

**Table 4:** The finite element analysis results of the three groups of lumbar vertebrae in flexion were compared - multiple samples Kruskal-Wallis test.

		23	24	25	P value	P^a^	P^b^	P^c^
Upper disc pressure(MPa)	IDP	0.46	0.51	0.57	0.161			
	AIDP	0.70	0.70	0.76	0.393			
	MIDP	0.36	0.47	0.52	0.050	1.0	0.050	0.294
	PIDP	0.28	0.31	0.44	0.180			
Lower disc pressure(MPa)	IDP	0.66	0.69	0.98	0.030	1.0	0.052	0.081
	AIDP	0.69	0.80	0.96	0.034	0.519	0.028	0.648
	MIDP	0.63	0.58	0.94	0.020	1.0	0.043	0.050
	PIDP	0.70	0.68	0.80	0.286			
Upper FJF(MPa)		0.05	0.08	0.03	0.643			
Lower FJF(MPa)		0.46	0.47	0.78	0.148			
ROM(°)		5.18	5.60	6.29	0.116

### 3.4 Compared the extensional moment of IDP, FJF and ROM on the extensional position

In extension, the von Meiss stress at the upper disc level of the transitional segment in the 23 PSV group was significantly increased than both the 24 and 25 PSV groups (*P* = 0.003). In the sub-region of the upper disc of 23 PSV, the stress of the anterior part was significantly greater than that at 25 PSV (*P* < 0.001), and the stress in the middle disc was higher than that of 24 and 25 PSV (*P* = 0.022, 0.002). Reversely, there was no significant difference in the stress at the lower disc of the transitional segment among the three groups (*P* = 0.381).

**Fig. 7:**
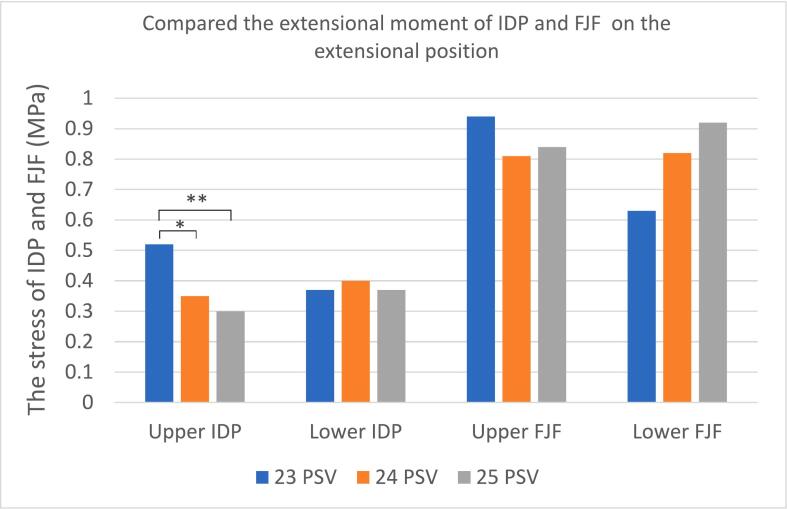
Comparison of IDP and FJF from upper to lower transitional lumbosacral segment among three groups on the extensional position. *P < 0.05, **P < 0.01.

**Fig. 8:**
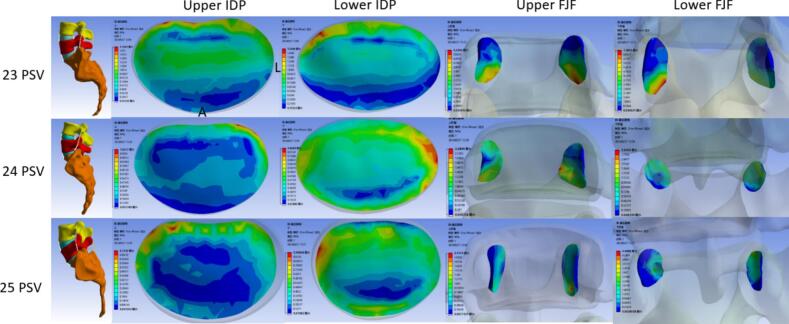
Stress nephogram of IDP and FJF from upper to lower transitional lumbosacral segment in three groups of lumbar vertebrae on the extensional position.

The stress of FJF at the upper segment of transitional segment in 23 PSV was higher than that of the other groups, but no difference among three groups both upper and lower segments (*P* = 0.423). As similar to FJF, the ROM in 23 PSV exhibited the largest value (3.28°), which was significantly larger than that of 25 PSV (1.40°) (*P* = 0.011). (Table. 5, Fig. 7, Fig. 8).

**Table 5:** The finite element analysis results of the three groups of lumbar vertebrae during extension were compared - multiple samples Kruskal-Wallis test.

		23	24	25	P value	P^a^	P^b^	P^c^
Upper disc pressure(MPa)	IDP	0.52	0.35	0.30	0.003	0.036	0.004	1.0
	AIDP	0.30	0.22	0.18	0.001	0.339	<0.001	0.081
	MIDP	0.56	0.30	0.28	0.002	0.022	0.002	1.0
	PIDP	0.60	0.48	0.48	0.036	0.084	0.070	1.0
Lower disc pressure(MPa)	IDP	0.37	0.40	0.37	0.381			
	AIDP	0.21	0.27	0.22	0.196			
	MIDP	0.30	0.33	0.37	0.441			
	PIDP	0.53	0.55	0.53	0.547			
Upper FJF(MPa)		0.94	0.81	0.84	0.423			
Lower FJF(MPa)		0.63	0.82	0.92	0.512			
ROM(°)		3.28	1.90	1.40	0.015	0.063	0.011	0.320

## 4 Discussion

Morphological changes in the spinal anatomy can alter body kinematics and stress transmission. LSTV is a common congenital variant of the lumbosacral junction and leads to early-onset spinal degeneration in young people. The Castellvi classification is a widely used system for categorizing LSTV based on morphological variations of the transverse processes, particularly the degree of fusion or articulation between the transverse processes of the lowest lumbar vertebra and the sacrum. The type of LSTV could significantly affect spinal biomechanics due to variations in structural morphology, such as the degree of fusion or articulation between the transitional vertebra and the sacrum. These morphological differences may alter load distribution, ROM, and stress patterns across adjacent spinal segments, potentially contributing to varying risks of degeneration or injury (Golubovsky et al., 2020; Mahato et al., 2019). Our study investigates the impact of purely numerical variations on the local intervertebral discs and facet joints at the Castellvi type I LSTVs.

This case-control matched study applied 3D finite element analysis to investigate the substantial biomechanical effects of LSTV number variants on adjacent discs and facet joints. The main findings were that the significant increase of stress on the upper disc of the transitional segment was only found in the 23 PSV (L5 complete sacralization) group during extension, while no such results in the 25 PSV (S1 complete lumbarization) group, and natural and flexion positions in all groups. Thus, we suggested only the 23 PSV sub-type of Castellvi type I LSTVs could aggravate the damage to the upper disc of the transitional segment, but not all sub-type variants, and the extension could be the potential factor leading to local segment degeneration.

In the neutral axial following loads state, the intervertebral disc stresses of the lower lumber spine were generally increased from L4 to S1 levels in three groups. The stress transmission of discs at the lumbo-sacral junction in patients with LSTV was similar to normal subjects, which was consistent with the reported stress changes of the normal curved lumbar spine in a scholar's study (Naserkhaki et al., 2016b). No difference in disc stress was found at the upper level of the transitional segment among the three groups. However, the disc stress at the lower level of the transitional segment in the 25 PSV group was significantly improved, especially in the middle (0.61 MPa) and posterior (0.74 MPa) parts of the disc, which were significantly higher than the 23 and 24 PSV groups. It is generally recognized that the number variations of spine can alter the physiological curvature of the lumbar spine. 23 PSV displays a relatively straight physiological curvature due to L5 complete sacralization, conversely S1 complete lumbarization results in a relatively larger curvature in 25 PSV with six lumbar vertebrae (Luo et al., 2024; Mahato, 2011). The increased lumbar curvature will lead the stress to transmit backward and fall in the middle and posterior part of the intervertebral disc through the more curved force line (Stott and Driscoll, 2023). In flexion, the significant difference in the von Meiss stress of the disc among the three groups was also only found in the lower intervertebral disc of the transitional segment. The stresses in the anterior (0.96 MPa) and middle (0.94 MPa) parts of the lower intervertebral disc in the 25 PSV group were significantly higher than that of 23 PSV (0.69 MPa, 0.63 MPa) during flexion, which were different with neutral position. Subjects with 25 PSV showed a bigger ROM (6.29°) compared to 23 and 24 PSV groups correlating to the increased curvature caused by six lumber vertebrae variations. The spine with 25 PSV showed more flexibility during flexing motion due to more bending curves. A previous study regarding FEA of the spine with different curvatures has found that hyper-lordotic spines exhibit a greater range of motion during flexion (Naserkhaki et al., 2016b). The increased flexion ROM of the 25 PSV would make the spinal transmission force line to move forward, thereby augmenting the strain in the anterior and middle part of the disc (Naserkhaki et al., 2016a).

During extension, the stress of the intervertebral disc at the upper level of the transitional segment was found a significant difference among the three groups, otherwise was no difference in the lower disc of the transitional segment, which was opposite to neutral and flexion positions. The von Meiss stress of the upper intervertebral disc in the 23 PSV group was significantly higher than that of the 24 and 25 PSV, regardless of comparison in the overall or each part of the disc. The spine with 23 PSV usually manifested as a small curvature compared to 24 and 25 PSV, which was similar to a Roussouly II type spine. In this type, the apex of lumbar curvature is located at the lumbar 4 level, and the stress is often concentrated at the apex level during extension (Menezes-Reis et al., 2016). Our findings regarding the horizontal stress increase in the upper disc of the transitional segment in 23 PSV were supported by other scholars' studies which reported more lumbar 4/5 levels of disc herniation were found in the Roussouly II spine (Chen et al., 2023; Menezes-Reis et al., 2016; Roussouly and Pinheiro-Franco, 2011). As for the range of motion, the spine with 23 PSV also showed a bigger ROM (3.28°) than 25 PSV (1.40°) during extension. This was different from the opinion of previous scholars who suggested that the straighter spine was more rigid and the spine with greater sagittal curvature had more flexible movement (Stott and Driscoll, 2023). The 23 PSV with a smaller curvature leaves more space for posterior appendages, while more bending lumbar spine will reduce the gap between spinous processes in the 25 PSV. This difference in posterior appendage space may explain our findings of ROM during extension. Our results were indirectly supported by a previous study that Baastrup's disease was easier to detect in patients with greater spinal curvature (Filippiadis et al., 2015).

The impact of numerical variants of Castellvi type I LSTVs on the development of intervertebral discs surrounding transitional segments was also explored in this study. In both the 23 PSV and 25 PSV groups of LSTV, the volumes at the upper disc of the transitional segment were larger than those at the lower levels. This trend was similar to the normal group and consistent with previous studies of disc spaces on imaging by Mahato (Mahato, 2011). The volumes of both the upper and lower discs of the transitional segment in 23 PSV and 25 PSV groups were larger than the normal group, however, the differences were not significant among them. In addition, the cartilage stresses at the upper and lower levels of facet joints surrounding transitional segment did not find significant differences among the three groups regardless of neutral, flexion and extension positions. This indicated that the number variation of Castellvi type I LSTVs had no substantial influence on the congenital development of intervertebral disc and cartilage stress of facet joints at the lumbosacral junction.

This study has some limitations. Firstly, this study focused on investigating the influence of pure numerical variation of Castellvi type I LSTVs on the biomechanical properties of adjacent discs and facet joints. Therefore, partially and completely fused transverse processes of LSTV were excluded to avoid the interference of transverse process pseudoarthrosis. We acknowledge that the Castellvi classification of transverse process variations is an important component and covers a broad scope of LSTV variations. We are working on exploring the impact of different Castellvi classifications of LSTV on lumbosacral segment biomechanics using 3D FEA in future work. These studies may provide a comprehensive understanding of LSTV-related biomechanics. Secondly, our study only established the finite element model of the local lumbo-sacral segments, but not the complete lumbar segment, which may restrict our findings to the generalized understanding of the biomechanics of whole lumbar segments. The behavior of the lumbar spine is influenced by interactions between multiple segments, and a model incorporating the entire lumbar spine could provide additional insights into compensatory mechanisms or load distribution patterns in the presence of LSTV. The influence of LSTV was not evaluated on the structures of the upper lumbar spine. Finally, the stress changes of pulposus and annulus fibrosus were not discussed between LSTV and normal subjects. Future work should build the finite element model of the whole spine to analyze the detailed impact of LSTV, and conduct a comprehensive study combined dynamic function assessments with varied real-world circumstances to develop a multi-dimensional understanding of the relationship between LSTV and lumbar degenerative changes.

## 5 Conclusion

In conclusion, the sub-types of LSTV number variation have different biomechanical stress effects on adjacent disc and facet joints at the transitional segment. Only the 23 PSV sub-type number variation of Castellvi type I LSTVs could exacerbate injury to the upper disc of the transitional segment, and posterior extension motion may be a potential factor in accelerating local segmental degeneration. This may give a further explanation and refinement to the current cognition of the impact of LSTV on the surrounding structures.

## CRediT authorship contribution statement

**Tongxin Zhu:** Writing – original draft, Software, Investigation, Funding acquisition. **Zhangyan Xu:** Investigation, Formal analysis. **Dan Liu:** Investigation, Data curation. **Wei Zeng:** Validation, Methodology. **Yongliang Pu:** Resources, Data curation. **HaiTao Yang:** Supervision, Conceptualization.

## Ethical approval

Institutional Review Board approval was obtained.

## Funding

The authors state that this work has not received any funding.

## Declaration of competing interest

The authors declare no competing interests.

## Data Availability

The data that has been used is confidential.
